# Serum Level and Activity of Butylcholinesterase: A Biomarker for Post-Stroke Dementia

**DOI:** 10.3390/jcm8111778

**Published:** 2019-10-24

**Authors:** Yi-Chun Chen, Wen-Hai Chou, Chiu-Ping Fang, Tung-Hsia Liu, Hsiao-Hui Tsou, Yun Wang, Yu-Li Liu

**Affiliations:** 1 Department of Neurology, Chang Gung Memorial Hospital Linkou Medical Center and College of Medicine, Chang Gung University, Taoyuan 333, Taiwan; ycchen.cgmh@gmail.com; 2 Center for Neuropsychiatric Research, National Health Research Institutes, Miaoli County 35053, Taiwaneikon@nhri.org.tw (C.-P.F.); wenliu@nhri.org.tw (T.-H.L.); 020120@nhri.edu.tw (Y.W.); 3 Division of Biostatistics and Bioinformatics, Institute of Population Health Sciences, National Health Research Institutes, Miaoli County 35053, Taiwan; 940903@nhri.org.tw; 4 Graduate Institute of Biostatistics, College of Public Health, China Medical University, Taichung 44047, Taiwan

**Keywords:** cholinergic system, stroke, dementia, biomarker, post-stroke dementia, cerebral infarction

## Abstract

Cholinergic neurotransmission regulates the immune response and inhibits cytokine release after stroke. The changes in the level/activity of blood cholinesterase (ChE) in patients with post-stroke dementia (PSD) are less known. This study aimed to examine post-stroke plasma acetylcholinesterase (AChE) and butylcholinesterase (BChE) and determine whether they are biomarkers for PSD. Thirty patients with PSD, 87 post-stroke patients without dementia (PSNoD), and 117 age- and gender-matched healthy controls were recruited. Missense genetic variants *AChE* rs1799806 and *BChE* rs1803274 were genotyped. The plasma AChE level did not differ between the PSD and PSNoD groups. However, BChE levels were significantly lower in the PSD than in the PSNoD group (3300.66 ± 515.35 vs 3855.74 ± 677.60 ng/mL, respectively; *p* = 0.0033). The activities of total ChE, BChE, and AChE were all lower in the PSD group (19,563.33 ± 4366.03, 7650.17 ± 1912.29, 11,913.17 ± 2992.42 mU/mL, respectively) than in the PSNoD group (23,579.08 ± 5251.55, 9077.72 ± 1727.28, and 14,501.36 ± 4197.17 mU/mL, respectively). When further adjusting for age and sex, significance remained in BChE level and activity and in total ChE activity. *BChE* rs1803274 was associated with reduced BChE activity, while *AChE* rs1799806 did not influence AChE activity. The level and activity of BChE, but not of AChE, were decreased in PSD patients and may therefore aid in PSD diagnosis.

## 1. Introduction

Stroke is a major cause of dementia, especially in the elderly [1,2]. Specifically, the one-year post-stroke dementia (PSD) rates after first-ever and recurrent stroke are approximately 10% and 30%, respectively [3]. Neuropathological studies into Alzheimer’s disease (AD) have shown that AD pathology is often concomitant with ischemic lesions in the elderly. For instance, evidence from the Nun Study showed that lacunes increase the risk of clinical expression of cognitive impairment more than 20 times during early Braak stages that were yet sufficient to produce dementia symptoms [4]. It follows that vascular insults enhance the clinical expression of AD pathology [5]. Vascular dementia is more prevalent in men and in populations affected by cerebral small-vessel disease, including Asians, Hispanics, and Africans [6,7].

The cholinergic system is distributed throughout the central nervous system [8], playing a role in neurotransmission and possibly mediating various neuroprotective functions [9]. Furthermore, cholinergic neurotransmission regulates the immune response during inflammation [10], in a pathway that involves nicotinic acetylcholine receptors (nAChRs) and inhibits peripheral cytokine release [11]. In particular, the efferent vagus nerve may release acetylcholine (Ach), which subsequently binds with macrophage receptors [12] and therefore may inhibit the release of inflammatory factors after stroke [12,13]. In addition to inflammation, excitotoxicity and energy failure following cerebral ischemia may also initiate intracellular death pathways and oxidative stress (OS). The structural and functional changes caused by OS led to cholinergic and other cellular damage. Cholinergic deficit further caused brain atrophy and finally resulted in cognitive impairment. Anticholinergic agents have been shown to regulate OS and alleviate apoptosis. Therefore, a key role in the pathogenesis of stroke and dementia may be attributed to OS [14].

Cholinergic dysfunction is related to vascular dementia [14]. Specifically, Ach levels in the cerebrospinal fluid are up to 49% lower in patients with small-vessel vascular dementia than in control patients, suggesting that cholinergic agents or cholinesterase inhibitors, both of which increase the Ach level, could be used to treat such patients [15]. One pilot study showed that acetylcholinesterase (AChE) inhibitor promoted functional recovery in elderly patients with cognitive impairment who had suffered stroke [16]. In a rat model of chronic ischemic, AChE activity was slightly decreased in the frontal cortex, but muscarinic acetylcholine receptor M1 was gradually depleted in cerebral cortex, striatum and hippocampus that are involved in cognitive function [17]. In addition, in stroke patients, reduced AChE activity was correlated with lower survival rate. Taken together, these results suggest that AChE dysfunction is a result of stroke rather than a cause for further neurodegeneration [13]. Moreover, AChE inhibitors exert no effect on stroke risk in dementia patients [18]. These observations suggest that Ach and AChE function may predict post-stroke neuronal damage. To date, only a few blood biomarkers for predicting PSD have been examined (e.g., D-amino acid oxidase [19], β-secretase [20], and inflammation markers [21,22,23]), and few studies have focused on changes in blood cholinesterase (ChE) levels and activity in patients with PSD [24]. Since plasma Ach is metabolized by AChE and butylcholinesterase (BChE), the present study examined the functional activities of these two enzymes in a population of patients with chronic ischemic stroke (IS).

## 2. Materials and Methods

### 2.1. Subjects

Patients with IS were enrolled from Chang Gung Memorial Hospital. All patients or their representatives provided informed consent to participate in this study. The study was carried out in accordance with the recommendations of Chang Gung Memorial Hospital Institutional Ethics Review Board (201600197B0) and the Research Ethics Committee, National Health Research Institutes (EC1051105-E-R1). Ischemic stroke and its subtypes were diagnosed and classified according to the patients’ clinical presentations and brain imaging. The inclusion criteria of stroke patients were (1) chronic stroke more than 30 days after the onset of stroke, and (2) stroke subtypes were limited to lacunar or atherothrombotic infarction to decrease the heterogeneity within the stroke groups. Lacunar infarction was diagnosed when the brainstem or subcortical hemispheric ischemia was <1.5 cm in diameter while atherothrombotic infarction was diagnosed when the ischemic lesion was >1.5 cm and arterial stenosis was >50% of any of the carotid/cerebral artery, as we previously reported [19]. Exclusion criteria of stroke patients included patients with cognitive impairment before stroke. Healthy control subjects with no history of stroke, neurodegenerative disease, overt clinical disease, or cancer were recruited randomly from the community. Patients with stroke who had no cognitive complaints were classified into the post-stroke without dementia (PSNoD) group after evaluation by two neurologists. PSD was diagnosed according to the requirements of the National Institute of Neurological Disorders and Stroke Association Internationale pour la Recherche et l’Enseignement en Neurosciences (NINDS-AIREN) criteria for probable vascular dementia [19,25]. The severity of cognition was basically evaluated by Clinical Dementia Rating (CDR) [26].

### 2.2. Clinical Information

Anthropometric data were collected from all participants. Hypertension (HTN) was defined based on the average of three independent measurements of systolic blood pressure ≥ 140 mmHg, diastolic blood pressure ≥ 90 mmHg, or based on the use of antihypertensive drugs. Diabetes mellitus (DM) was defined based on World Health Organization (WHO) criteria [27]. Alcohol drinking was defined as drinking amount more than 210 g per week. Smoking was defined as former (had smoked at least 100 cigarettes in lifetime, but currently not) or current smoking (have smoked 100 cigarettes in lifetime and currently smoke daily or nondaily) [28]. Because strong anticholinergic drugs may influence the risk of dementia, we recorded the participants’ prescription during blood collection to evaluate the exposure to the drugs that were listed in the prior study [29].

### 2.3. Sample Preparation for Activities Assay, Protein Assay, and Genotyping Assay

Ten mL whole blood were collated with ethylenediaminetetraacetic acid (EDTA) blood collection tube and centrifuged with 1500× *g* at 4 °C for 15 min and processed for isolation of plasma within 2 h of blood collection, then frozen at −80 °C, and stored until analysis. The average time for the frozen sample stored was 4.09 ± 1.19 years. Genomic DNA was extracted from the buffy coat of whole blood lymphocyte pellets using the Gentra Puregen Blood kit (QIAGEN Sciences, Germantown, MD, USA).

### 2.4. Cholinesterase Activities Assay

The total ChE activity was assayed using the DetectX^®^ acetylcholinesterase fluorescent activity kit (Arbor assays, Ann Arbor, MI, USA). Both AChE and BChE inside the plasma metabolize the substrate acetylthiocholine iodide from the kit to produce thiocholine and acetic acid. The thiocholine then reacts with the proprietary, non-fluorescent molecule, ThioStar^®^ to yield a thiocholine-ThioStar fluorescent complex, which can be read at 510 nm in a fluorescent plate reader, with excitation at 390 nm. The assay procedure was carried out according to the protocol provided with the kit. The plasma was diluted 1000-fold using the 1× Assay Buffer. Next, 100 μL of the diluted plasma sample or AChE standard were pipetted into each well in the plate, with the 100 µL 1× Assay Buffer as zero concentration; 50 μL of the Reaction Mix was then added to each well and pipetted several times to ensure adequate mixing. The reaction mixture was incubated at room temperature for 20 min and then read at 510 nm, with excitation at 370 nm. The standards were analyzed with duplication, and samples were analyzed with one point in one plate during experiment.

Butylcholinesterase activity was assayed using the DetectX^®^ butyrylcholinesterase fluorescent activity kit (Arbor assays, USA), which uses a similar principle, but with a butyrylthiocholine iodide substrate, which reacts with BChE only. AChE activity was determined by subtracting the BChE activity from the total ChE activity, measured in terms of fluorescence intensity (AChE activity = total ChE activity—BChE activity).

### 2.5. Plasma AChE and BChE Protein Assay

AChE and BChE plasma proteins were measured using commercially available quantitative ELISA kits (R&D Systems, Minneapolis, MN, USA), which employ the quantitative sandwich enzyme immunoassay technique. Antibodies specific to human AChE and BChE were pre-coated onto a microplate. The test plasma sample required 2-fold dilution using 1× Calibrator Diluent RD5-26 due to the matrix effect of AChE, and a 1000-fold dilution with 1× Calibrator Diluent RD5P in the case of BChE. Next, 50 μL of Assay Diluent RD1-63 for AChE and 100 μL of Assay Diluent RD1-21 for BChE were added to each well; 50 μL of standard or sample was then pipetted into each well and incubated for 2 h on a horizontal orbital microplate shaker. After four washes using Wash Buffer, 200 μL of Human AChE or BChE Conjugate was added to each well and incubated for 2 h at room temperature on a horizontal shaker. Four more washes with Wash Buffer were performed, and 200 μL of Substrate Solution was then added to each well and incubated for 30 min at room temperature on the benchtop, with light avoidance. Color development was stopped by adding 50 μL of Stop Solution. The intensity of the color was immediately measured using a microplate reader at 450 nm. The wavelength was corrected by subtracting readings at 570 nm from readings at 450 nm. The standards were analyzed with duplication, and samples were analyzed with one point in one plate during experiment.

### 2.6. Functional Single Nucleotide Polymorphisms in AChE, BChE, and APOE

There are 19 single nucleotide polymorphisms (SNPs) for *AChE* [30]. Only rs1799806 on exon 6 of *AChE* was associated with activity changes. The AChE activity of homozygote Pro/Pro genotype was significantly lower than Arg/Arg genotypes. The SNP rs1803274 on exon 5 is the most studied K-variant in *BChE* has 33% reduced plasma BChE activity [31]. To identify whether genetic polymorphisms within *AChE* or *BChE* could predict the functional activity of the enzymes, missense SNPs rs1799806 on exon 6 of *AChE* and rs1803274 on exon 5 of *BChE* were selected and genotyped using TaqMan SNP genotyping assay (Thermo Fisher Scientific, Waltham, MA, USA). The SNPs of *APOE*, rs429358 and rs7412, were also genotyped with TaqMan SNP genotyping assay (Thermo Fisher, USA). For all subjects, 10 ng of genomic DNA was used for genotyping, according to the protocols provided with the TaqMan Genotyping Master Mix (ThermoFisher, USA; for rs1799806, rs429358 and rs7412) and the TaqMan GTXpress Master Mix (ThermoFisher, USA; for rs1803274). Ten ng genomic DNA, 20× TaqMan genotyping probe, and 2× Master Mix were well mixed in a total 10 µL reaction volume, and the real-time PCR reaction was performed on StepOnePlus system (ThermoFisher, Scientific, Waltham, MA, USA). For SNPs genotyped with TaqMan Genotyping Master Mix, the conditions is starting at 95 °C for 10 min on enzyme activation stage, and flowing the 50 cycles of denaturation at 95 °C for 15 s, and annealing/extension at 60 °C for 1 min. For TaqMan GTXpress Master Mix, the conditions is starting at 95 °C for 20 s on enzyme activation stage, and flowing the 50 cycles of denaturation at 95 °C for 1 s, and annealing /extension at 60 °C for 20 s.

### 2.7. Statistics

The continuous data of patients with IS were compared with those of healthy controls using the Mann–Whitney U test. Categorical data were compared using the chi-square test. Spearman’s correlation analysis was used to test the associations between AChE activity and age. The normal distribution of continuous data was tested using normality tests based on the Shapiro–Wilk test. When further adjusting for age and sex, general linear model (GLM) procedure was performed to compare PSD patients and PSNoD patients in continuous data (such as 30-day MRS, post-stroke time, plasma AChE level, plasma BChE level, ChE activity, BChE activity, AChE activity). Logistic regression analysis was used to compare PSD patients and PSNoD patients in categorical data (such as HTN positive/negative, DM positive/negative, *APOE* e4 carrier positive/negative) when further adjusting for age and sex. Two-way ANOVA was used to assess the association of plasma BChE activity between groups (IS versus healthy controls) and functional genetic polymorphism (such as *BChE* rs1803274). All these analyses were performed using SAS software, Version 9.4 (SAS Institute, Inc., Cary, NC, USA). The statistical plots were further calculated and plotted using GraphPad Prism 5 (GraphPad Software, San Diego, CA, USA).

## 3. Results

### 3.1. Characteristics of Patients with Ischemic Stroke

In total, 117 patients with IS and 117 age- and sex-matched healthy controls were recruited (Table 1). The average age of these patients was 65 years, and 63% were male. Years of education were fewer in the stroke patients (7.39 ± 4.30 years) than in the control group (12.50 ± 4.73 years, *p* = 0.04). Both HTN and DM were more prevalent in patients with stroke than in the controls (chi-square test: *p* = 0.01 and 0.0006, respectively). Proportion of smoking was higher in stroke patients than in the controls (*p* = 0.009). Proportions of alcohol drinking and exposure to anticholinergic drugs were similar between the patients with stroke and the controls. The average time elapsed since the patients’ stroke was 2.31 ± 2.96 years, and 25.64% of the patients with stroke also had PSD. With regards to ChE function, plasma AChE and BChE levels did not differ significantly between patients with IS and healthy controls, neither did the activity of ChE, BChE, and AChE differ between the groups. Furthermore, there was no difference in the distribution of the *APOE* e4 carrier.

With regard to the presence or absence of dementia in patients with IS, the PSD group was older than the PSNoD group (*p* < 0.0001) (Table 2). Years of education were significantly fewer in the PSD (3.69 ± 4.05 years) than in the PSNoD group (8.74 ± 3.56 years, *p* < 0.0001). Both HTN and DM showed greater prevalence in patients with PSD than in those without. However, after adjustment for age and sex, the difference was not significant. Proportions of alcohol drinking and smoking were similar between the PSD and PSNoD groups. Both the PSD and PSNoD groups showed mild 30-day disability, but the PSD group had a higher 30-day MRS score (1.63 ± 1.38) than the PSNoD group (0.76 ± 0.75, *p* = 0.003). The stroke subtypes were not different between PSD and PSNoD. PSD patients tended to have higher exposure rate to anticholinergic drugs (36.67%) than the PSNoD patients (12.64%, *p* = 0.004). However, exposure to these drugs did not influence levels and activities of AChE and BChE. The plasma AChE level did not differ between the PSD and PSNoD groups. However, BChE level was significantly lower in the PSD group than in the PSNoD group. The activities of total ChE, BChE, and AChE were all lower in the PSD group than in the PSNoD group, and the differences in BChE level and activity and total ChE activity remained significant after adjustment for age and sex. The adjust variables age and years of education are highly correlated (Spearman’s *r* = −0.527, *p* < 0.0001), that is, both variables had multicollinearity and the GLM model should include only one of the two variables [32].

### 3.2. Decreased Plasma AChE and BChE Activity with Aging in Patients with and without Stroke

**Figure 1:**
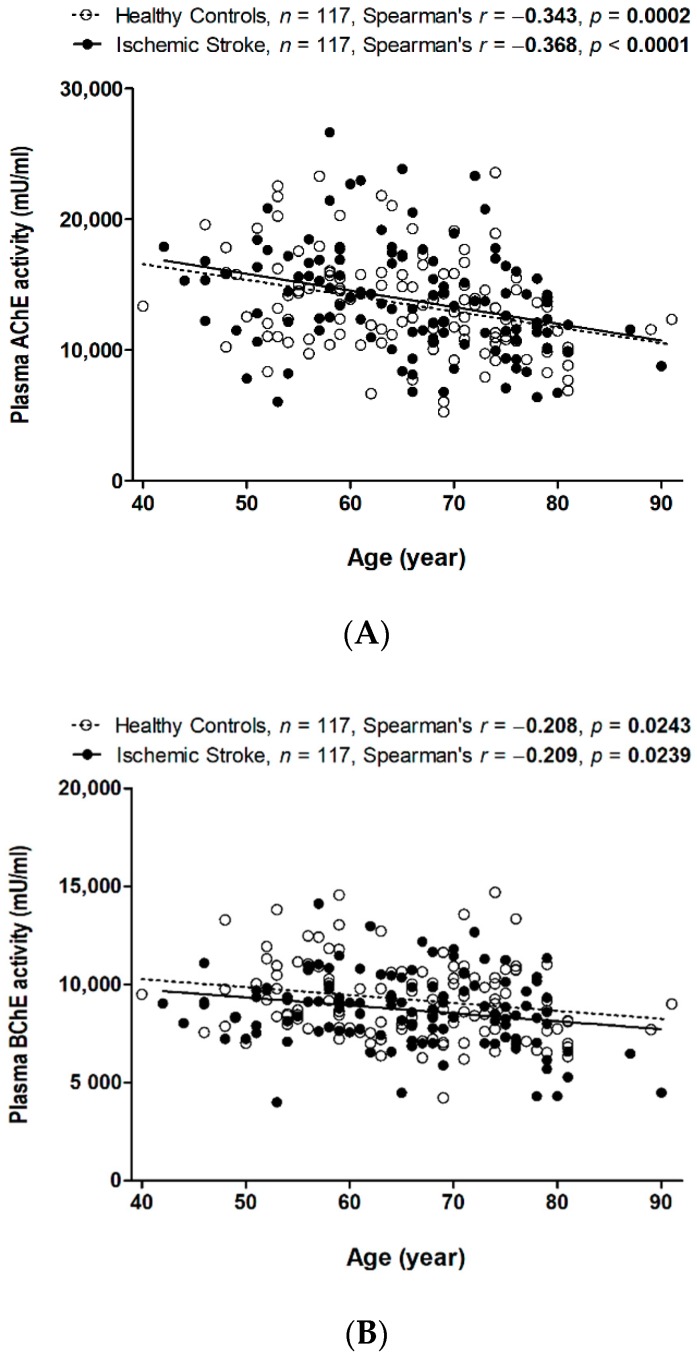
Spearman’s correlation between (**A**) AChE and age and (**B**) BChE activity and age when the patients were separated into healthy controls and patients with IS.

Spearman’s correlation revealed that plasma AChE activity was negatively correlated with age in both healthy controls (*r* = −0.343, *p* = 0.0002) and stroke patients (*r* = −0.368, *p* < 0.0001) (Figure 1A). Plasma BChE activity was also borderline negatively correlated with age in both healthy controls and stroke patients (*r* = −0.208, *p* = 0.0243 and *r* = −0.2088, *p* = 0.0239, respectively) (Figure 1B).

### 3.3. Genetic Influences of Functional SNPs on AChE and BChE in Ischemic Stroke

The selected SNPs of the *AChE* (rs1799806), *BChE* (K variant, rs1803274), and *APOE* (rs429358 and rs7412) genes were all in Hardy–Weinberg equilibrium (*p* > 0.05). None of the four SNPs were associated with stroke risk in multiple tests (Table 3). One functional genetic polymorphism, BChE rs1803274, reduced BChE activity (mU/mL) (*p* = 0.027) (Table 4). Specifically, it predicted roughly 22% less BChE activity in healthy controls carrying the CT genotype than in those carrying the CC genotype, while BChE activity was around 3% lower in patients with ischemia carrying the CT genotype than in those carrying the CC genotype. In contrast, AChE rs1799806 did not influence AChE activity, and APOE did not influence either AChE or BChE activity.

### 3.4. Optimal Cut-off Point Calculated by Receiver Operating Characteristic Curve (ROC) Analysis for PSD Diagnosis

**Figure 2:**
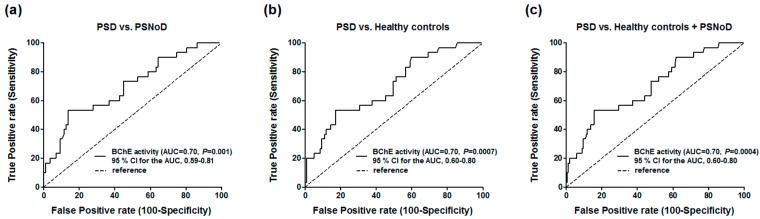
ROC curve with plasma BChE activity between the post-stroke dementia (PSD) and (**a**) the post-stroke patients without dementia (PSNoD), (**b**) healthy controls or (**c**) healthy controls + PSNoD. AUC, Area under the curve. CI, Confidence interval.

In the area under the curve (AUC)/ROC analysis, the level of plasma BChE activity was significantly correlated with PSD, comparing to healthy controls (AUC = 0.70; 95% confidence interval (CI), 0.60–0.80; *p* = 0.0007), PSNoD (AUC = 0.70; 95% CI, 0.59–0.81; *p* =0.001), or controls + PSNoD (AUC = 0.70; 95% CI, 0.60–0.80; *p* = 0.0004) (Figure 2). The optimal cut-off value of plasma BChE activity for the diagnosis of PSD versus healthy controls is 7423 mU/mL (sensitivity = 53.3%, specificity = 82.9%).

## 4. Discussion

AChE and BChE are two major enzymes regulating the plasma level of Ach, which binds a muscarinic receptor on macrophages to regulate the inflammatory response after stroke [12]. The present study involved a population with chronic ischemic stroke that had an average duration of two years, and it found that BChE level and activity, as well as total ChE activity, were significantly lower in the PSD group, independent of age and sex. In contrast, no significant difference was found between the PSD and PSNoD groups in terms of plasma AChE level and activity. This finding was the first to suggest that BChE level and activity may be a marker for PSD. An unstable, functional genetic polymorphism *BChE* variant (rs1803274) reduced BChE activity, while *AChE* rs1799806 did not influence AChE activity, and *APOE* had no influence on either AChE or BChE activity. 

The pathological process of post-stroke neurodegeneration involves inflammation [33,34], which has been associated with cholinergic neurons [12]. Specifically, the efferent vagus nerve may release Ach, which then binds with muscarinic receptors on macrophages and inhibits the release of inflammatory factors such as tumor necrosis factor-α and other cytokines, thus modulating the immunological responses [12,13]. Therefore, circulating AChE activity may be an indicator for the inflammatory response [13].

The cholinergic system may be a therapeutic target influencing recovery after brain injury [35]. For example, stimulation of nAChRs may directly regulate microglia and macrophages to limit inflammation in the brain [36,37]. In a rat model of ischemic stroke, nAChR antagonists aggravated neural damage in the cerebral cortex, suggesting that endogenous Ach may regulate the pathogenic events of ischemic injury [38]. Conversely, an agonist of cholinergic neurotransmission functioning through allosteric modulators via α7 nAChR conferred endogenous neuroprotection against ischemic injury [39].

Various cholinesterase inhibitors approved for the treatment of AD have shown modest cognitive benefits in patients with vascular dementia, with no consistent benefit regarding global change outcomes. As such, the clinical significance of these drugs is unclear [40,41]. Specifically, high-dose donepezil for 6 months improved cognition function and activities of daily living in patients with mild or moderate vascular cognitive impairment [42]. In patients with mild cognitive impairment following stroke, rivastigmine has inconsistent efficacy and may cause side effects [43]. Similarly, galantamine showed inconsistent benefits [44,45].

Although there is a high risk of PSD in chronic stroke patients, no effective biomarker has been established for PSD [46]. The secondary neurodegenerative changes have been reported as neuroimaging biomarkers for cognitive impairment [47]. For blood biomarkers, only a few have been examined, such as D-amino acid oxidase [19], β-secretase [20], inflammation markers [21,22,23], and homocysteine [48]. We previously reported that the sensitivity and specificity of plasma D-amino acid oxidase for the diagnosis of PSD were 75% and 88.7%, respectively [19]. The use of these biomarkers for diagnosis of PSD is still limited by their specificity and sensitivity.

The present study was the first to suggest that the activity and level of serum BChE, but not of AChE, may be markers for PSD. However, because the AUC of the ROC curve was only about 0.7 with inadequate power to separate PSD and healthy controls (sensitivity = 53.3%, specificity = 82.9%), utilizing plasma BChE activity alone may not exert sufficient diagnostic value for PSD. BChE activity is 20-fold lower than that of AChE with regard to Ach hydrolyzation, and BChE accounts for up to 90% of total serum ChE [49]. Although its function has not been definitively clarified, BChE is a predictor for two-year major cardiac events, as well as for Parkinson’s disease and related dementia [50,51]. The present study suggested that the unstable *BChE* variant rs1803274 (K genotype) is associated with reduced activity [13]. However, we did not find that this variant was associated with stroke susceptibility in the present study. Differences in sampling timeline, stroke subtypes, sample number, and ethnicity may cause these disparities.

Although the present study did provide evidence indicating that BChE level and activity are markers for PSD, it had several limitations. Firstly, we did not measure Ach, which would have directly illuminated the role of the cholinergic system in PSD. Secondly, PSD was diagnosed by clinical presentation, which may have led to underdiagnosis in patients with very mild executive dysfunction or over-diagnosis in those with depression, as we previously mentioned [19,52]. Finally, the number of participants was relatively small. As such, the results of the present study need to be replicated before serum BChE can be viewed as an independent predictor of PSD. Prospective longitudinal studies with comprehensive neuropsychological tests are needed to strengthen these findings.

## 5. Conclusions

The level and activity of BChE, but not of AChE, were decreased in PSD patients and may therefore aid in PSD diagnosis. An unstable, functional genetic polymorphism *BChE* variant (rs1803274) reduced BChE activity, while *AChE* rs1799806 did not influence AChE activity.

## Figures and Tables

**Table 1:** The association analyses between ischemic stroke patients and healthy controls.

Variable	Healthy Controls	Ischemic Stroke	*p*-Value
	*n*, Mean ± SD	*n*, Mean ± SD	
Age	117, 65.48 ± 10.07	117, 65.60 ± 10.11	0.89
Gender			1.00
Female	43 (36.75%)	43 (36.75%)	
Male	74 (63.25%)	74 (63.25%)	
Education duration (years)	4, 12.50 ± 4.73	109, 7.39 ± 4.30	**0.040**
HTN			**0.010**
No	50 (43.48%)	32 (27.35%)	
Yes	65 (56.52%)	85 (72.65%)	
DM			**0.0006**
No	90 (78.26%)	67 (57.26%)	
Yes	25 (21.74%)	50 (42.74%)	
Alcohol drinking			0.42
No	103 (88.03%)	97 (84.35%)	
Yes	14 (11.97%)	18 (15.65%)	
Smoking			**0.009**
No	100 (85.47%)	82 (71.30%)	
Yes	17 (14.53%)	33 (28.70%)	
CDR	-	117, 0.32 ± 0.61	-
30-day MRS	-	117, 0.98 ± 1.02	-
Post-stroke dementia (%)	-	30, 25.64%	-
Stroke subtypes			-
Lacunar infarction	-	75 (64.10%)	
Atherothrombotic infarction	-	42 (35.90%)	
Anticholinergic drugs			0.21
No	102 (87.18%)	95 (81.20%)	
Yes	15 (12.82%)	22 (18.80%)	
Plasma AChE (pg/mL)	67, 1271.41 ± 292.19	73, 1245.73 ± 404.16	0.28
Plasma BChE (ng/mL)	76, 3822.30 ± 782.08	76, 3731.58 ± 682.61	0.42
ChE activity (mU/mL)	117, 22,723.16 ± 4974.9	117, 22,549.4 ± 5321	0.84
BChE activity (mU/mL)	117, 9240.96 ± 1979.02	117, 8711.68 ± 1875.64	0.12
AChE activity (mU/mL)	117, 13,482.21 ± 3738.36	117, 13,837.72 ± 4072.74	0.47
*APOE* e4 carrier	18 (16.51%)	12 (11.43%)	0.28

HTN: Hypertension. DM: Diabetes mellitus. CDR: Clinical Dementia Ranting. MRS: Modified Rankin scale. AChE: Acetylcholinesterase. BChE: Butylcholinesterase. ChE: Cholinesterase. SD: Standard Deviation. Mann–Whitney U test in 2 groups for continuous data and Chi-square test for categorical data. The bold font of *p*-Value means *p* < 0.05.

**Table 2:** Association analyses among ischemic stroke patients with or without dementia.

Variable	PSNoD	PSD	Unadjusted/Adjusted *p*-Value
	*n*, Mean ± SD	*n*, Mean ± SD	
Age	87, 62.98 ± 9.23	30, 73.20 ± 8.68	**<0.0001**
Sex			0.67
Female	31 (35.63%)	12 (40.00%)	
Male	56 (64.37%)	18 (60.00%)	
Education (years)	80, 8.74 ± 3.56	29, 3.69 ± 4.05	**<0.0001/<0.0001 ^1^**
HTN			**0.0001**/0.96 ^1^
No	32 (36.78%)	0 (0.00%)	
Yes	55 (63.22%)	30 (100.00%)	
DM			**0.027**/0.14 ^1^
No	55 (63.22%)	12 (40.00%)	
Yes	32 (36.78%)	18 (60.00%)	
Alcohol drinking			0.86/0.25 ^1^
No	72 (84.71%)	25 (83.33%)	
Yes	13 (15.29%)	5 (16.67%)	
Smoking			0.45/0.84 ^1^
No	59 (69.41%)	23 (76.67%)	
Yes	26 (30.59%)	7 (23.33%)	
30- day MRS	87, 0.76 ± 0.75	30, 1.63 ± 1.38	**0.0013/0.003 ^1^**
Post-stroke time (years)	87, 2.15 ± 2.98	30, 2.79 ± 2.90	0.17/0.84 ^1^
CDR	87, 0.03 ± 0.12	30, 1.15 ± 0.70	**<0.0001/<0.0001 ^1^**
Stroke subtypes			0.32 / 0.42 ^1^
Lacunar infarction	58 (66.67%)	17 (56.67%)	
Atherothrombotic infarction	29 (33.33%)	13 (43.33%)	
Anticholinergic drugs			**0.004**
No	76 (87.36%)	19 (63.33%)	
Yes	11 (12.64%)	11 (36.67%)	
Plasma AChE (pg/mL)	56, 1279.02 ± 428.62	17, 1136.07 ± 294.71	0.28/0.16 ^2^
Plasma BChE (ng/mL)	59, 3855.74 ± 677.60	17, 3300.66 ± 515.35	**0.0033/0.025 ^2^**
ChE activity (mU/mL)	87, 23,579.08 ± 5251.55	30, 19,563.33 ± 4366.03	**0.0008/0.014 ^2^**
BChE activity (mU/mL)	87, 9077.72 ± 1727.28	30, 7650.17 ± 1912.29	**0.0012/0.002 ^2^**
AChE activity (mU/mL)	87, 14,501.36 ± 4197.17	30, 11,913.17 ± 2992.42	**0.0029**/0.08 ^2^
*APOE* e4 carrier	10 (12.99%)	2 (7.14%)	0.41/0.48 ^1^

HTN: hypertension. MRS: Modified Rankin scale. CDR: Clinical Dementia Ranting. PSD: Post-stroke dementia. PSNoD: Post-stroke without dementia. SD: Standard Deviation. ^1^ Adjusted *p*-value-1: General linear model or logistic regression analysis, adjusted for age and sex. ^2^ Adjusted *p*-value-2: General linear model, adjusted for age, sex, and additionally for exposure to anticholinergic drugs when examine levels and activities of AChE, BChE, and ChE. Chi-square test for categorical data. Mann–Whitney U test for continuous data. The bold font of *p*-Value means *p* < 0.05.

**Table 3:** *AChE*, *BChE* and *APOE* associated with ischemic stroke and healthy controls.

Gene/SNP	Healthy Controls		Ischemic Stroke		*p*-Value
	*n*	(%)	*n*	(%)	
*AChE*/rs1799806					0.037
GG	93	(85.32%)	75	(71.43%)	
GC	16	(14.68%)	29	(27.62%)	
CC	0	(0.00%)	1	(0.95%)	
*BChE*/rs1803274					0.063
CC	92	(84.40%)	77	(73.33%)	
CT	16	(14.68%)	28	(26.67%)	
TT	1	(0.92%)	0	(0.00%)	
*APOE* type					0.242
ε3/ε3	77	(70.64%)	84	(80.00%)	
ε3/ε4	14	(12.84%)	12	(11.43%)	
ε2/ε3	14	(12.84%)	9	(8.57%)	
ε4/ε4	3	(2.75%)	0	(0.00%)	
ε1/ε3 or ε2/ε4	1	(0.92%)	0	(0.00%)	
*APOE* ε4					0.28
non-carriers	91	(83.49%)	93	(88.57%)	
carriers	18	(16.51%)	12	(11.43%)	

Chi-square test was used.

**Table 4:** The association of plasma BChE activity (mU/mL) between the groups and rs1803274.

Group	rs1803274 Genotype	*n*	Mean ± SD	*p*-Value	
				Group	rs1803274
Healthy controls	CC	92	9416.40 ± 1884.43	0.098	0.027
	CT	16	7721.56 ±1582.06		
	TT	1	7753.00		
Ischemic Stroke	CC	77	8708.75 ± 1708.44		
	CT	28	8473.50 ± 1993.10		
	TT	0	-		

Two-way ANOVA was used.
